# Conduction delays across the specialized conduction system of the heart: Revisiting atrioventricular node (AVN) and Purkinje-ventricular junction (PVJ) delays

**DOI:** 10.3389/fcvm.2023.1158480

**Published:** 2023-04-19

**Authors:** Bum-Rak Choi, Ohad Ziv, Guy Salama

**Affiliations:** ^1^ Department of Medicine, Rhode Island Hospital and Brown University, Providence, RI, United States; ^2^ Department of Medicine, Heart and Vascular Institute, University of Pittsburgh, Pittsburgh, PA, United States

**Keywords:** step-delay-Wenckebach periodicities, voltage-sensitive dyes-high resolution optical mapping, Purkinje-ventricular delays, mechanisms of atrioventricular delay, Purkinje-papillary activation

## Abstract

**Background and significance:**

The specialized conduction system (SCS) of the heart was extensively studied to understand the synchronization of atrial and ventricular contractions, the large atrial to His bundle (A-H) delay through the atrioventricular node (AVN), and delays between Purkinje (P) and ventricular (V) depolarization at distinct junctions (J), PVJs. Here, we use optical mapping of perfused rabbit hearts to revisit the mechanism that explains A-H delay and the role of a passive electrotonic step-delay at the boundary between atria and the AVN. We further visualize how the P anatomy controls papillary activation and valve closure before ventricular activation.

**Methods:**

Rabbit hearts were perfused with a bolus (100–200 µl) of a voltage-sensitive dye (di4ANEPPS), blebbistatin (10–20 µM for 20 min) then the right atrial appendage and ventricular free-wall were cut to expose the AVN, P fibers (PFs), the septum, papillary muscles, and the endocardium. Fluorescence images were focused on a CMOS camera (SciMedia) captured at 1K-5 K frames/s from 100 × 100 pixels.

**Results:**

AP propagation across the AVN-His (A-H) exhibits distinct patterns of delay and conduction blocks during S1–S2 stimulation. Refractory periods were 81 ± 9, 90 ± 21, 185 ± 15 ms for Atrial, AVN, and His, respectively. A large delay (>40 ms) occurs between atrial and AVN activation that increased during rapid atrial pacing contributing to the development of Wenckebach periodicity followed by delays within the AVN through slow or blocked conduction. The temporal resolution of the camera allowed us to identify PVJs by detecting doublets of AP upstrokes. PVJ delays were heterogeneous, fastest in PVJ that immediately trigger ventricular APs (3.4 ± 0.8 ms) and slow in regions where PF appear insulated from the neighboring ventricular myocytes (7.8 ± 2.4 ms). Insulated PF along papillary muscles conducted APs (>2 m/s), then triggered papillary muscle APs (<1 m/s), followed by APs firing of septum and endocardium. The anatomy of PFs and PVJs produced activation patterns that control the sequence of contractions ensuring that papillary contractions close the tricuspid valve 2–5 ms before right ventricular contractions.

**Conclusions:**

The specialized conduction system can be accessed optically to investigate the electrical properties of the AVN, PVJ and activation patterns in physiological and pathological conditions.

## Introduction

The cardiac conduction system (CCS) is a network of specialized cardiac myocytes for impulse generation and transmission to the heart muscle for synchronous conduction. Historically, the discovery of CCS of the heart was made by anatomists who had few research tools at their disposal, namely the microscope but who had with remarkable powers of observation (1). The anatomical structure and the electrophysiology of the CCS are closely linked to its function which is to synchronize atrial contractions and after a substantial delay produce the rapid, synchronous contractions of the ventricular chambers. Various pathological conditions such as inherited diseases, ischemia/infarction, infection, high blood pressure, cardiomyopathies, aging, surgery, or drug-induced cardiotoxicity can lead to dysfunction in CCS, which can cause fatal arrhythmia or sudden death.

Electrical impulses or action potentials (APs) from the atria converge to the atrioventricular node (AVN) which is a sliver of small cells, a few millimeters in length, located in the posterior wall of the right atrium immediately behind the tricuspid valve. In healthy hearts, the AVN serves as the only pathway that links electrically the atria to the ventricles, a station to delay the APs by over 100 msec and produce sufficient current to trigger APs in the His bundle. Downstream from the His-bundle, the structure of the Purkinje network and the electrical coupling between Purkinje and Ventricular myocytes occurs at specialized Junctions. Purkinje network and the location of Purkinje-Ventricular Junctions (PVJs) are key elements for the stability and resilience CCS and the synchronization of atrial and ventricular contractions (2).

Although the main function of CCS is to deliver impulses for the rapid propagation of APs and to synchronize cardiac function, conduction delays in the CCS play critical roles in fine tuning atrial and ventricular contractions. The AVN receives APs from surrounding transitional cells from the fast and slow pathways and delays AP propagation to the His bundle and Purkinje network to ensure that atrial contractions precede ventricular contractions (3). The His bundle after the central fibrous body is divided into right and left bundle branches which are insulated maintain rapid conduction and accurate timing of ventricular contractions. For example, the initial right bundle branch of Purkinje fibers connects to the base of the papillary muscle before reaching the apex or free wall of the right ventricle. This anatomical feature of PFs branching earlier to the papillary muscles may be designed to initiate the closure of the valves before ventricular contractions to prevent regurgitation of blood. The Purkinje network also creates conduction delay, particularly in PVJ and the conduction through PVJ is thought to be discontinuous with significant conduction delay (4, 5), which can serve to overcome source-sink mismatch between the Purkinje myocytes and the large number of ventricular myocytes surrounding them.

Despite the importance of proper conduction delays in CCS, the location of delays, conduction blocks, and their rate-dependent dynamics are not fully understood. Advances in molecular and developmental biology allow reconstruction of detailed anatomical variations within different cell types throughout the AV node (6–9) and PVJs (10–12) but functional correlation is still a matter of conjecture. Previous mapping studies with multi-electrodes and optical mapping techniques lacked spatial and temporal resolution to resolve complex conduction pathways and delays across the AVN and PVJs. Here, we re-examine the impulse propagation through the AVN and PVJs using optical mapping at higher spatial (100 × 100 pixels) and temporal resolution (up to 5,000 f/s) with a CMOS camera [instead of a 16 × 16 photodiode array (13)] and investigated the location and distribution of conduction delays and conduction blocks.

## Methods

### Heart preparation

New Zealand white rabbits (*n* = 11, male and female, 5–12 months old, were euthanized with buprenorphene [0.03 mg/kg IM (intramuscular injection)], acepromazine (0.5 mg kg^−1^ IM), xylazene (15 mg kg^−1^ IM), ketamine (60 mg kg^−1^ IM), pentothal (35 mg kg^−1^ IV), and heparin (200 U kg^−1^). This investigation conformed to the current Guide for Care and Use of Laboratory Animals published by the National Institutes of Health (revised 2011). Hearts were excised from the chest and perfused in a Langendorff perfusion apparatus (Radnoti LLC, Covina CA). Blebbistatin (10–20 μmol L^−1^) was perfused to reduce movement artifact (14). For AVN optical mapping, a linear cut was made on the right atrial appendage and pinned down to expose the AV node area as previously described (15). Most studies of the AVN with intracellular microelectrodes, dissect and isolate the AVN from the heart and bathe the preparation with oxygenated buffer, insuring spatial heterogeneities of tissue oxygenation. Here, we use a preparation that maintains the coronary perfusion of the atria and ventricles as well as the AVN and the CCS. Moreover, measurements of the AVN and the detection of conduction blocks in the AV node, or His bundle were made in physiologically stable preparation. To map the AVN and PVJs, a linear cut was made along the posterior aspect of the heart, base to apex to expose the right ventricular septum and endocardium The major arteries and most coronary vessels were then systematically tied with silk sutures to seal the leaks of perfusate and re-establish a physiological perfusion pressure (≥70 mmHg) which was monitored at the aorta. Electrical stimuli were delivered through a concentric bipolar electrode that was located on the interatrial septum slightly above the triangle of Koch, and which triggered AP propagation mainly through the fast pathway input to the AVN. The experimental protocol to study PF and ventricular activation patterns followed a systematic sequence of mapping impulse propagation through right bundle branch. Initially, the base of the papillary muscle arising from the RV septum was focused on the camera to map PVJs and the field-of-view was moved to the endocardium of the RV free wall to focus on intact free-running Purkinje fibers. The PVJ delays were mapped within 1 h of the dissection to optically view PVJs to avoid possible ischemic injury.

### Optical mapping of impulse propagation through AV node and PV junction

The optical apparatus was described previously (16). Voltage sensitive dye, di-4 ANEPPS (Invitrogen), was delivered through the bubble trap located over the perfusion cannula (20 µl of 2 mM di-4 ANEPPS stock solution in DMSO). Fluorescence images from the triangle of Koch were focused on a CMOS camera (100 × 100 pixels, Ultima-L, Scimedia, Japan). The field of view was set to 5 mm × 5 mm (50 µm × 50 µm spatial resolution, per pixel) to locate and record signals from the AVN and 4 mm × 4 mm (40 µm × 40 µm spatial resolution, per pixel) from the PVJs (using 25 mm f0.95 Navitar video lens). The sampling rate of optical recordings was set to 1 K for AVN mapping and 2 K or 5 K f/s for PV junction mapping. Data were analyzed with custom-built software using Interactive Data Language (ITT Visual Information Solutions, Boulder, CO, USA (17). The fluorescence recordings (F) were filtered using non-linear bilateral filter (13 ms window size) and the first derivatives (dF/dt) were calculated by convolution of gaussian first derivative kernel (13 ms window size). The maximum dF/dt were mapped for AP propagation. As described in (13, 18, 19), the fluorescence signal recorded from the single pixel at the AV junction is the sum of multiple cells in the region, including cells from deeper layers (depth of the field of view 200 µm). We mapped the propagation of each upstroke and correlated optically identified AP upstrokes with signals from bipolar electrogram recordings (15).

## Results

### Dynamics of AV nodal conduction delay and locations of AV block

**Figure 1:**
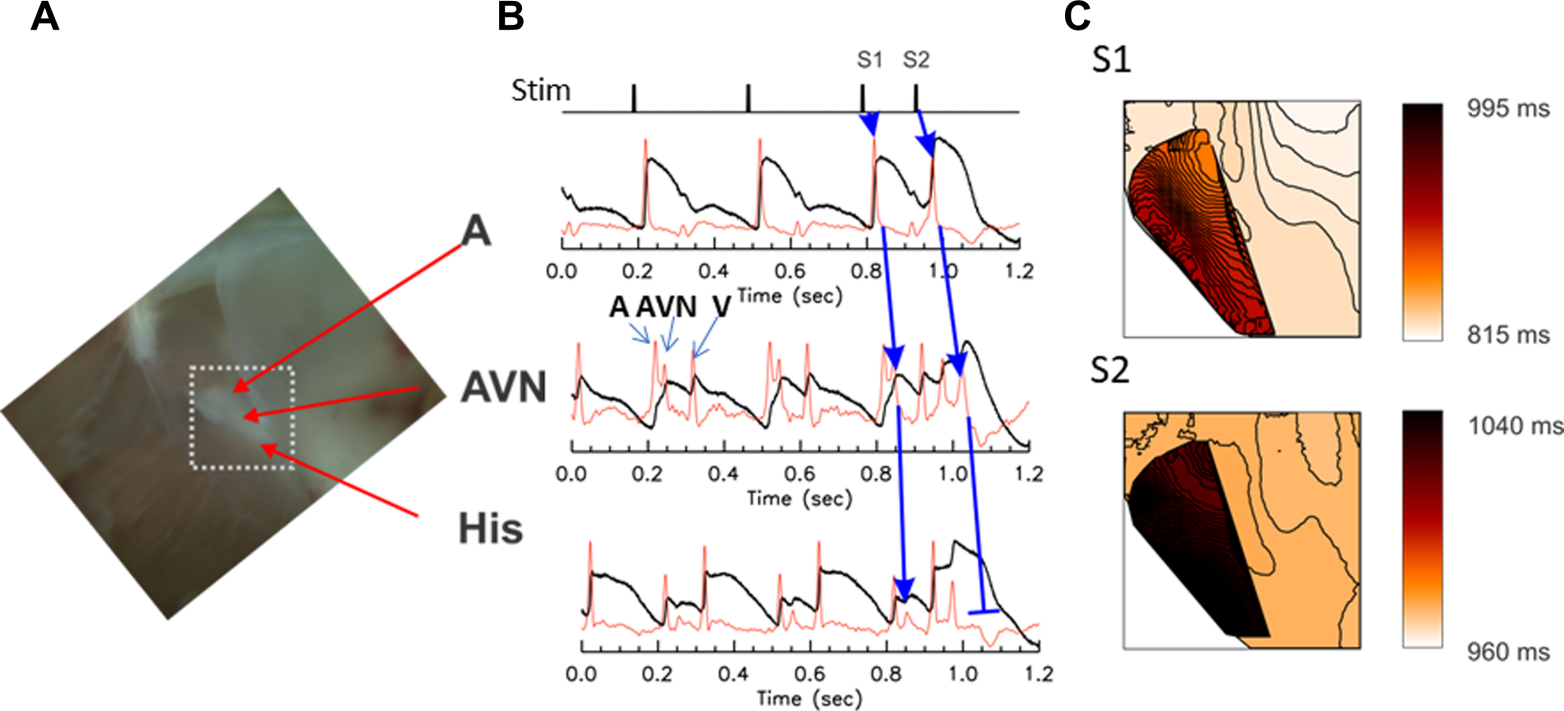
Optical mapping of conduction delay through the AV node. The AV node is a three-dimensional structure consisting of multiple layers of myocytes with different electrical properties and delays in firing; as a result, three distinct upstrokes are detected from each pixel, in the optical voltage-recordings from the AV node region. (**A**) A photo of the Triangle of Koch. The mapped area is marked with a white dotted box (5 s mm × 5 s mm). (**B**) Representative optical mapping traces recorded from the AVN region of a rabbit heart under S1–S2 stimulation protocol. The first derivative (red traces) shows three distinct upstrokes associated with atrial (A), nodal/His (AVN, blue arrows), and ventricular (V) AP. The atrial tissue was paced at S1–S2 (basic CL = 300 ms followed by S2 at 140 ms). The upstroke of the AVN AP appeared substantially later in the S2 beat than the S1 beat (see the middle trace) and propagated toward His-bundle with a diminishing magnitude of the AP upstroke compared tothe S1 beat (blue arrows for S2 beat), indicating decremental conduction. (**C**) Activation maps of atrial and nodal-His upstrokes. The atrial and nodal upstrokes were mapped separately, then are superimposed to visualize the overlap of the atrial and nodal tissues. Isochronal lines are drawn every 1 ms interval. The conduction velocity of AVN propagation of control beat was 0.101 mm/ms while the conduction of premature S2 beat in the AV node was slightly slower (0.08 mm/ms) and died out in the His bundle, in this example. A movie of AV nodal conduction of sinus beat, and S1 and S2 beats are provided as the Supplementary Material respectively as Supplementary Movies S1, S2.

AV block is a partial or complete interruption of impulse transmission from the atria to the ventricles. The conduction block can happen in the atria, AV node, and/or His bundle. To study AV block, the rabbit atrial tissue was paced using S1S2 protocol from the interatrial septum above the AV node which triggers AP propagation through the fast pathway. The propagation of premature beats through AV nodal region was recorded using optical mapping as described in Methods. Previous studies of the rabbit AV node by optical mapping showed that multiple action potential (AP) upstrokes were detected in fluorescence recordings from a single pixel, representing atrial, AV nodal, and ventricular action potential upstrokes, originating from different depths of the preparation (18). Figure 1 shows a typical optical mapping experiment from the anatomical region shown in panel A. Panel B is a recording of trace from the rabbit AVN region. The first derivatives of optical traces exhibit 3 distinct upstrokes per sinus rhythm beat (panel B). The mapping of the first two upstrokes shows independent propagation patterns corresponding to atrial and nodal AP propagation (panel C).

During rapid pacing, two sites of conduction block were routinely observed. The first conduction block occurred at the cycle length close to the ventricular refractoriness. Sample traces are shown in Figure 1B during S1–S2 pacing that led to the AVN block. A detailed examination of AP upstrokes during an AVN block revealed that atrial to AVN conduction was intact (blue arrows from A to AVN traces). However, the conduction from the AVN to the His bundle was decremental, diminishing its amplitude in the His bundle area and failed to elicit ventricular APs. Panel C shows the activation maps of S1 and S2 beats (Supplementary Movies S1, S2) for the S1 and the S2 impulse. The darker color map of AVN propagation of S2 beat indicates that the step delay between A and AVN increased during the S2 beat and propagated slowly toward the His region. However, APs failed to propagate in the His region (panel B), indicating that the His bundle AP is in its refractory period.

**Figure 2:**
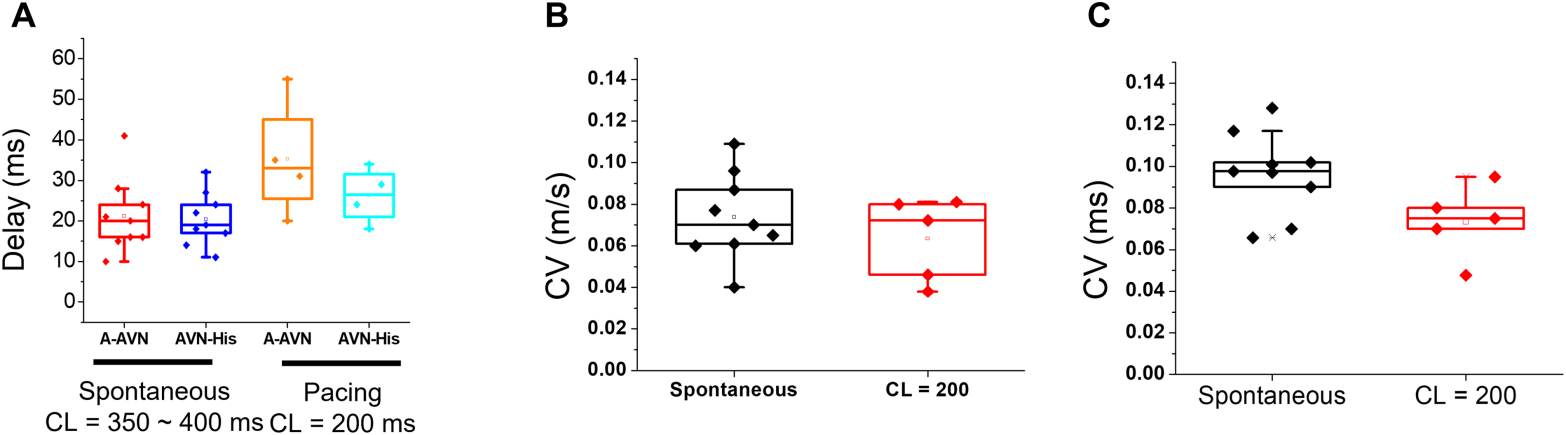
Conduction delay and velocity measurements across the AVN during spontaneous rhythm and pacing. (**A**) AV- delay of spontaneous and pacing at a CL (cycle length) = 200 ms. The step-delay between the atrial and the AV node APs (A-AVN) is responsible for a major AVN delay. (**B,C**) CVs of AVN conduction were measured using two methods, local vector using a 5 × 5 window (**B**, 0.074 ± 0.021 m/s for the spontaneous beat and 0.063 ± 0.02 m/s for the paced beat at 200 ms CL) or along the line from the initial AVN AP to the His bundle area (**C**, 0.096 ± 0.019 ms for the spontaneous beat and 0.074 ± 0.017 m/s for the paced beat at 200 ms CL).

In Figure 2, panels A–C show a summary of AV nodal delays and conduction velocities during sinus rhythm and pacing at 200 ms. We found that the AV delay has two components, a step delay between the atrium and the first AVN AP (A-AVN), and the delay by slow propagation from AVN to His (AVN-His, see panel A). The premature S2 beat is prolonged mainly due to the A-AVN delay (panel A orange vs. cyan box plots). Although CVs of S2 beat are slower (from 0.096 to 0.074 m/s, panel B and C), the propagation from AVN to His is brief occurring within several mm, which creates only small conduction delay.

**Figure 3:**
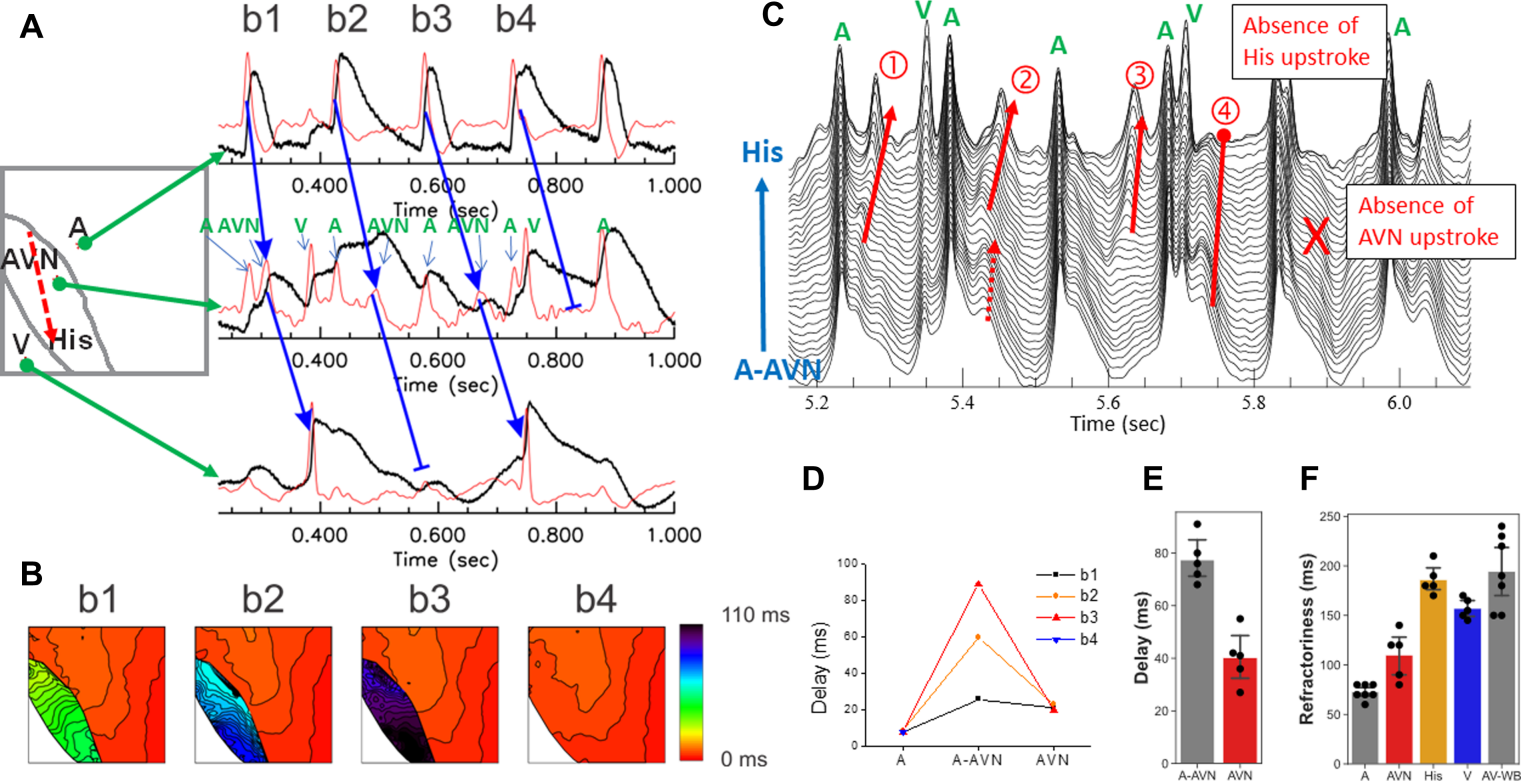
Two distinct locations of AV block. (**A**) Sample traces of rapid pacing at CL = 150 ms from the SA node region. In this example, the trace shows a 2:1 AV block and the conduction block occurred at two different locations (beat 1 and beat 3 conducted while beat 2 and beat 4 were blocked, see arrows). (**B**) Activation maps of b1–b4 beats. AV nodal signals were present in b1, b2, and b3 but absent in b4, indicating that the AV block of b2 occurred between the AVN to His pathway while the AV block of b4 occurred between the atrium and the AVN. The darker color of AVN maps in b2 and b3 indicate an increase of the step-delay between the atrium and the AVN occurred before the block. (**C**) Overlapped traces from the AV nodal region. The first derivative traces from the atrial to the His locations are overlapped to visualize AV nodal propagation. The red arrows indicate AV nodal propagation. AV nodal delay increases progressively from beat 1–3 but beat 4 failed at His and absence of AVN signal in this beat. (**D**) AV-delay of beat 1–beat 4. Most of the AVN delay was caused by the step delays between the atrium and the AVN (A-AVN, 20–89 ms) while the delay within the AVN to His bundle remains relatively stable in this episode (20–23 ms). (**E**) The longest AV-delay of during Wenckebach pacing. The step delay accounts for 69 ± 12 ms while the longest AVN to His bundle was 46 ± 9 ms (*n* = 5 hearts). (**F**) The refractory periods of the atrial, AVN, AVN-His, and V tissues. The refractory period of the AVN measured by S1–S1 protocol is close to that of the atrial tissue while the refractory period of the AVN-His is close to the ventricular refractory period, indicating that the initial AVN block occurs due to the refractoriness in the His bundle. However, with continuous pacing with increasingly shorter CL, the His/ventricular refractory period caused a complex Wenckebach conduction pattern associated with increasing AVN-delays and conduction blocks between the atrium and the AVN, and the AVN and the His bundle (a movie of WB conduction is provided in the Supplementary Material, as Supplementary Movie S3). A, atrial; AVN, AV node; AVN-His, AV nodal to His bundle; V, ventricular refractoriness; AV-WB, Wenckebach cycle length.

Persistent rapid pacing using S1-S1 protocol caused complex patterns of conduction blocks at multiple locations. Sample traces of S1-S1 pacing at 150 ms CL are shown in Figure 3. The first beat (beat 1) shows a normal conduction with all the AP upstrokes of A, AVN, and ventricular APs. Activation maps of A and AVN in panel B show a similar pattern of normal AV conduction, seen in Figure 1C. However, the second beat (beat 2) shows an absence of His and ventricular APs, suggesting that this conduction block occurred between the AVN and the His bundle region (beat 2 in Figure 3A) as seen during S2 pacing (Figure 1B). The third beat (beat 3) recovered with a normal AV conduction (blue arrow in panel A and activation map in panel B). The fourth beat (beat 4) exhibited a conduction block between the atrium and the AVN as indicated by the absence of AVN upstroke (beat 4 in panel A and 4th map in panel B), indicating that the atrial AP failed to evoke an AVN APs due to the refractoriness of the AVN. The waterfall plot of dF/dt traces from the AVN region in panel C show beat-to-beat changes in conduction pattern with increasing delay (beat 1–3) and AVN blocks at the two different locations (beat 4–5).

The rapid pacing of the atrial tissue caused wide range of AVN conduction delays due to (i) increased time to evoke AVN AP by the atrial tissue (a step delay between atrial and AVN AP), (ii) increased conduction time within the AVN toward His bundle due to slow conduction in the AVN. Beat-to-beat dynamics of AV conduction delays are shown in panel D. From beat 1 to beat 4, the step delay between A and AVN increased progressively from 26 to 89 ms while conduction delay between the AVN and the His bundle remained relatively stable before the AVN failed to evoke an AP in beat 4. The stable conduction between the AVN and the His bundle may be due to an increase in step-delay that causes longer diastolic intervals of premature beats in the AVN, which ensures sufficient recovery time for conduction. Panel E and F are summaries of the longest AV delay and the refractory periods obtained from a rapid pacing protocol (*n* = 5 hearts). The refractory periods of the AVN were close to the atrial tissue while the AVN-His refractory period is close to that of the ventricular tissue. During rapid pacing, the longer step-delay, and the slow conduction in the AVN, result in conduction blocks at multiple levels, between the atrium and the AV node, and between the AV node and the His bundle which cause complex AV conduction delays, exhibiting Wenckebach phenomena at much longer CL than the AVN refractory period (panel D, AV-WB, and Supplementary Movie S3).

### Optical mapping of Purkinje network reveals a step-delay between Purkinje and ventricular tissues

**Figure 4:**
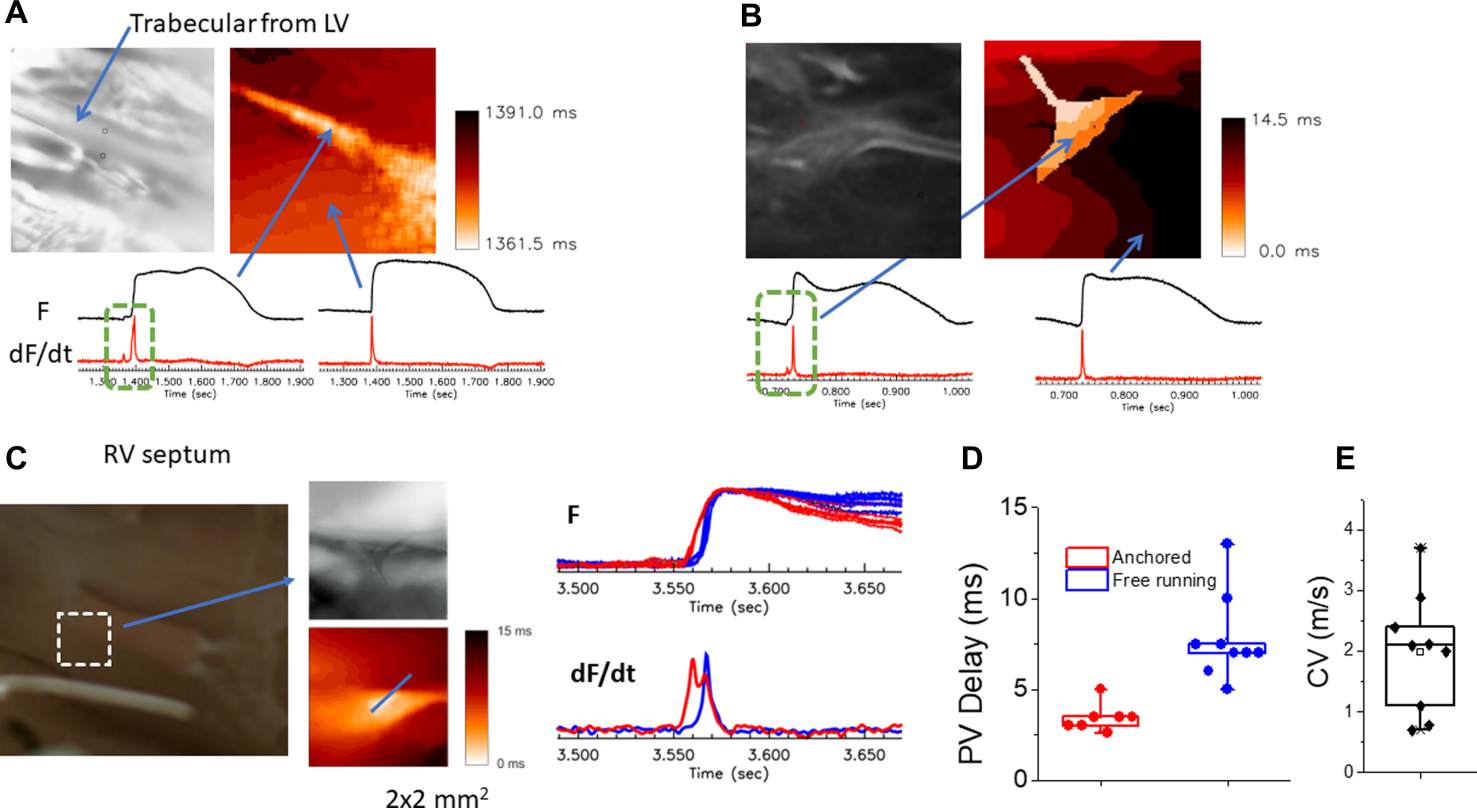
Detection of conduction delay through PVJs. (**A**) Two distinct upstrokes on optical mapping traces in Purkinje/endocardial region. The image on the left shows the location of mapping and trabecular muscle in the center. The optical mapping trace in one side of the trabecular muscle showed two distinct upstrokes (dotted green box), an initial small upstroke followed by a large upstroke (right panel, Supplementary Movie S4). The rest of regions in the field of view did not show two distinct upstrokes (the right trace). (**B**) Sample traces recorded over a free-running Purkinje fiber that is anchored to the trabecular muscles. Two distinct upstrokes are visible over the free-running Purkinje fiber (the left trace). The superimposed activation map shows early excitation representing the free-running Purkinje fiber (right panel, Supplementary Movie S5). (**C**) AP propagation toward papillary muscle (PM) of the right ventricle septum. High resolution mapping shows that free running Purkinje fibers anchor and activate at the base of the PM. The sample traces showed two distinct peaks; the free-running Purkinje bundle (red) compared to the rest of PM and endocardium (blue), indicating that the PM AP is triggered early by the Purkinje network (bottom right panel, Supplementary Movie S6). (**D**) Summary of conduction delay across PVJs. Purkinje-ventricular delay measured from the time delay between the two upstrokes varied from 2 to 13 ms (*n* = 8 hearts, *n* = 16 locations), most likely due to variability of Purkinje fiber insulation from the ventricular myocardium. (**E**) CV along the Purkinje fiber. Purkinje fiber CVs (1.97 ± 0.99 m/s) were measured from *n* = 9 locations selected for their linear propagation for at least 1-mm, without branching.

Typical optical mapping traces from the trabecular muscle, free-running Purkinje and papillary muscle are shown in Figure 4. Like recordings from the AV nodal region, multiple upstrokes (dF/dt) were seen from the single pixels in the region of trabecular muscle, free-running Purkinje, and papillary muscle regions. Under sinus rhythm, a tiny additional upstroke is seen prior to a large upstroke with a step-delay (panel A, marked with dotted green box). The activation maps of both upstrokes and its movie (Supplementary Movie S4) show that the first upstroke propagates along one side of the trabecular muscle followed by activation of the entire trabecular muscle and ventricular endocardium. This example illustrates that AP propagation through the specialized conduction system can be visualized through mapping of individual upstrokes identified by the first derivatives of the fluorescence recordings.

AP propagation through free-running Purkinje fibers (false tendon) can also be visualized with optical mapping. Panel B shows an example of optical mapping traces from the free-running Purkinje network found in the RV apex. Again, the trace from a single pixel over the Purkinje network exhibits two upstrokes (green box in panel B). The superimposed maps of individual upstrokes showed that the free-running Purkinje fibers are activated earlier and then trigger ventricular APs (Supplementary Movie S5).

Overall, the conduction between Purkinje and ventricular myocardium exhibits a wide range of step delay from 2 to 13 ms (panel D) and Purkinje fiber CV from 0.7 to 3.7 m/s (panel F). Importantly, ventricular activation patterns are not always in line with Purkinje activation patterns, consistent with Purkinje fibers being insulated from the endocardium and that their coupling with ventricular myocytes is limited to certain areas. The anatomical organization of the PFs plays a key role in the sequence of activation of papillary muscles before the activation of the ventricular chambers by 2–5 ms. As a result, the closure of the valves occurs before ventricular activation to secure the direction of blood flow out of the RV and LV arteries and the hemodynamic functions of the heart.

The papillary muscles in RV septum are connected to atrioventricular valves and during systole, papillary muscles contract in a timely manner to prevent inversion or prolapse of these valves. In Figure 4C, Purkinje fibers are anchored to the papillary muscle in the rabbit RV septum at the base and high-resolution optical mapping showed two upstrokes during activation (Supplementary Movie S6).

**Figure 5:**
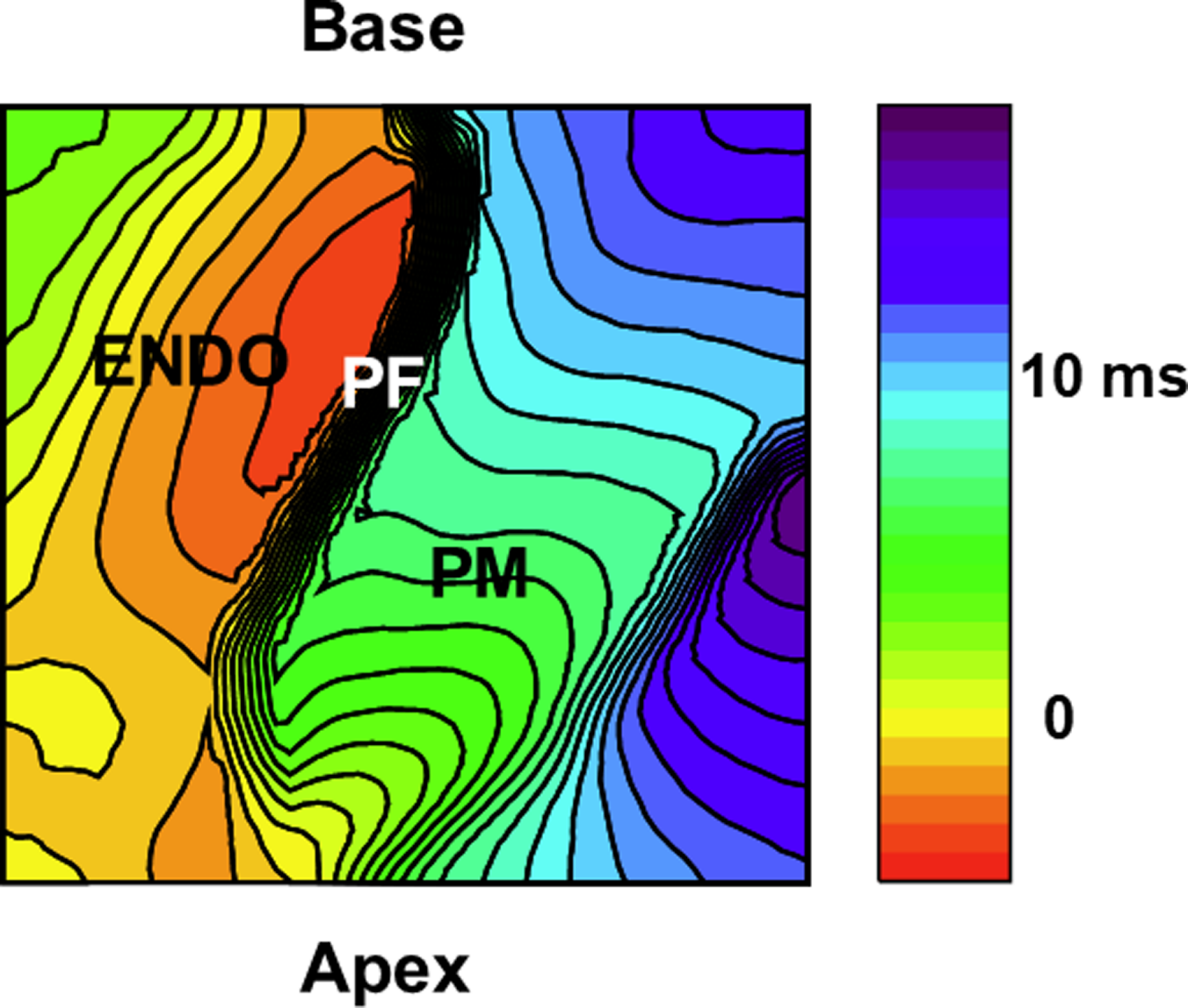
Activation patterns from purkinje-papillary-myocardium. Image of the papillary muscles from the RV septum from a 5 mm × 5 mm region. Activation map of the papillary muscle (PM) under sinus rhythm. An example of activation isochrones (1 msec apart) is shown for a right ventricular papillary muscle in a perfused rabbit heart. A fast wave of depolarization appears first along the PFs lying along the papillary (base to apex), followed by the firing of papillary APs before the spread of electrical activity along the endocardium (ENDO). The Supplementary Movie S7 depicts the sequence of activation demonstrating a wave of PF depolarization followed by the PM depolarization preceding the activation of the endocardium (ENDO) by 2–5 msec.

Figure 5 shows the activation patterns from a RV papillary muscle. Activation is first initiated in Purkinje fibers that quickly propagates along the one side of the papillary muscle, followed by a slower propagation along the papillary muscle then propagation of the endocardium (Figure 5; Supplementary Movie S7). This example illustrates that the connection of Purkinje fibers guarantees early activation of papillary muscles to avoid inversion of the atrioventricular valves during systole.

**Figure 6:**
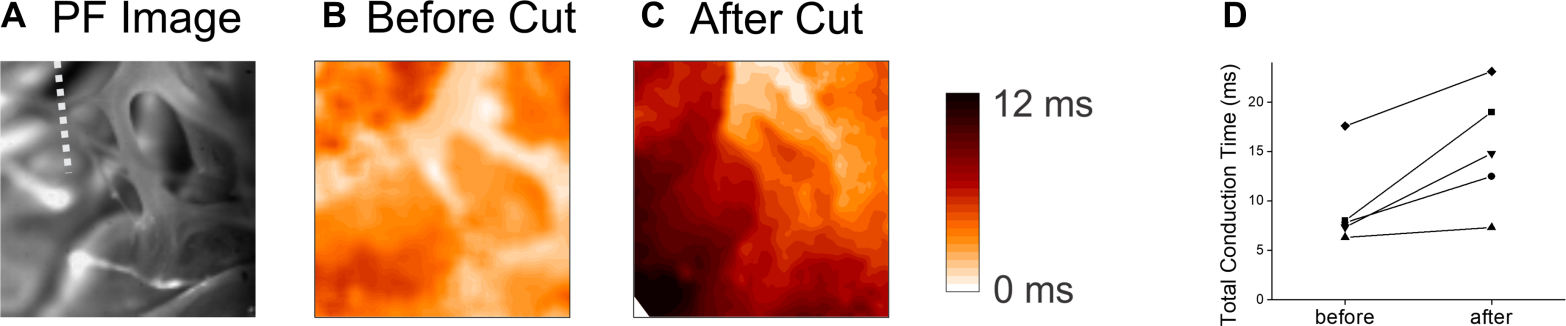
Endocardial activation time before and after cutting the free-running purkinje fibers. (**A**) Image of the PF network on the RV apex. The white dotted line indicates the location of cut. (**B**) Activation map before the cut. The activation of the endocardium in the field of view (4 mm × 4 mm) occurred within a short period of time (Supplementary Movie S8). (**C**) Activation map after cutting a PF branch indicated in panel **B**. After cutting, the activation pattern changed to a simpler unidirectional propagation (Supplementary Movie S9). (**D**) Summary of total activation time in the field of view (4 mm × 4 mm) before and after cutting a major PF branch. **E,F** panels are associated with the activation maps before and after the PF cut, respectively.

PF network is composed of multiple branches and hubs to provide resilience through redundancy. We further investigated how local branches influence the activation pattern of endocardium by cutting off free-running PF in the field of view. Figure 6 shows an example of PF branches from RV apex region (panel A) and corresponding activation pattern (panel B, Supplementary Movie S8), which shows synchronous activation). After cutting off a PF branch marked with a white dotted line in Panel A, the activation pattern was changed to show a propagating wave from the top to the bottom (panel C, Supplementary Movie S9). Cutting a PF branch increased the total activation time by 5.9 ± 3.6 ms (*n* = 5, panel D).

## Discussion

The specialized CCS generates heart rhythm and distribute electrical signals for the timely contraction of heart muscle. The anatomical and functional features of CCS underlie a resilient network of conduction for transmitting electrical signals and proper conduction delays in CCS are essential to achieve synchronous contractions. In this study, we optically mapped impulse propagation through the AV node, His bundle, and the PVJ. Conduction delays and refractoriness are uniquely determined in each anatomical location. Specifically, delays in the fast pathway of the AV node and the PVJs are localized in a small area (step-delays) that can be modulated under physiological conditions for efficient contraction of the heart muscle.

### Conduction through of the AV node

In healthy hearts, the AVN serves as the only pathway that links electrically the atria to the ventricles, as a station to delay the action potentials by over 100 msec and produces sufficient current to trigger APs in the His bundle. The cause for the delay between the electrical propagation of APs from the right atrium to the His bundle (A-H) has been the subject of considerable investigation as well as speculation (8, 20).

Immunohistology images using multiple markers (Cx43, Cx40, neurofilament, Nav, … etc.) (7, 21) revealed the existence of specialized cell types from the coronary sinus to the penetrating His bundle through the inferior nodal extension. The compact or AVN is located above the inferior nodal extension (INE) to form the fast pathway (6). The AVN delay therefore is thought to occur due to slow propagation across the compact AVN in the fast pathway as well as slow propagation through INE in the slow pathway. Our high-resolution mapping shows that the compact AVN is indeed responsible for the AVN delay through the fast pathway, in line with previous immune-histological, and functional mapping studies. However, our results show several new findings; (1) The AV delays are composed of two components including a delay between the atrial and nodal cells and a delay within the AVN caused by slow conduction, (2) the rate dependent prolongation of the AV delay through the fast pathway is mainly produced by a step-delay between the atrial and nodal cells, (3) the refractoriness of the AVN and the input from the fast pathway are not longer than atrial tissue as previously thought, (4) AV blocks occur at two locations in normal hearts including the His bundle and between the atrial and the AVN cells. The conduction block occurs first in the His bundle while AVN can still generate APs that are decremental to the His bundle, and a much shorter CL is required to cause the block in the AVN. Importantly, our data demonstrated that the first location of AP upstroke in AVN varies depending on the pacing CL. When CL is long (in the physiological HR), APs in the AVN start in the proximal area close to the entering excitation wave of atrial APs. However, during rapid pacing, the initial location of the AP upstroke moves into the center of the AVN and is associated with a much longer AVN-delay. Conduction velocity from the AVN to the His bundle is relatively stable in our recordings, even during Wenckebach periodicities, raising questions against the conventional AVN-delay mechanisms which posits that the delay is mainly due to slow conduction through the AVN.

### Location and mechanisms of the AV nodal delay

The large A-H delay has been attributed to a slow conduction velocity across the AVN caused by (a) the small cell size, (b) the connective tissue surrounding the AVN cells, (c) the slow rise of AP upstrokes, consistent with the lack of functional voltage-gated sodium channels, (d) a depolarized resting membrane potential (∼ −60 mV) and e) low conduction intercellular coupling between the AVN cells due to diminished gap-junction expression. This narrative is used to explain the A-H delay in medical school lectures (e.g., Shubham Tripathi King's College UK, lectures from 2018-present) and textbooks, despite substantial challenges to its validity (22–25). For this reason, the teaching of the mechanisms responsible for the AVN delay should be revisited and corrected. For example, in rabbit hearts, the AVN is ∼1.5 mm long and has a delay of about 120 ms, hence if propagation is continuous, the conduction velocity (CV) would have to be as slow as 0.0125 m/s which is well below the estimated threshold for stable propagation of 0.05 m/s (26).

Our data indicate that a significant delay already occurs between the atrial input AP and the first AV nodal AP upstroke. This observation is not new, as numerous studies indicated that the majority of AVN delay is localized in a narrow region at the entrance of the AVN. Hoffman et al., made use of a roving intracellular microelectrode to systematically measure APs along the length of rabbit AVNs using a fixed electrode on the atrium, as a reference (27). Measurements of the time delay between a fixed atrial electrode and the roving microelectrode indicted that “almost all the A-V delay is localized in a narrow zone extending the full width of the A-V node ∼1 mm across and located near the junction of the atrium and the A-V node” (27). The data emphasized that the delay was not uniformly distributed along the AVN but was localized in a zone of 0.25 mm at the junction between the atrium and the AVN with CV ranging from 0.05 to 0.02 m/s (at or below the threshold for stable propagation) (27). In a seminal study, Rosenblueth described a unique behavior of the AVN where the faster the rate of atrial stimulation the longer the AVN delay, until a threshold rate is reached to produce failure of ventricular activation and resulting in stable patterns of A-V transmission or Wenckebach cycles (28). The increase of A-H delay with increasing rate of atrial pacing, was tentatively attributed to “decremental conduction” where CV within the AVN is reduced with faster atrial stimulation (29). An explanation based on “decremental conduction” would predict that in the AVN, the AP refractory period increases rather than decreases with increasing rate. Rosenblueth argued that the V nodal conduction delay and Wenckebach periodicity of AV transmission cannot be due to overall decremental conduction within the AV node and proposed that a step-delay, caused by a special element or layer of the AV nodal tissue (28). The findings from the Hoffman and Rosenblueth studies are congruent, one based on experimental evidence in rabbit the other in canine AVN, respectively.

The step-delay mechanism was further supported by experimental and modeling studies of AVN propagation. Studies of human AVN attributed Wenckebach periodicity to a step-delay mechanism (30, 31), and intracellular microelectrode studies on rabbit AVN indicated that the increase in AVN delay with decreasing cycle length could not be explained by decremental conduction but a step-delay located centrally in the AVN (32). Mathematical models of AVN propagation and Wenckebach behavior included step-delays localized within the node (33, 34). These studies expressed the compelling requirement of a step-delay mechanism despite speculation about the nature of and precise location of this component of AV delay. Images of AVN propagation using voltage sensitive dyes in perfused rabbit AVN revealed that the step delay occurred at the boundary between the atrium and the node and was most likely caused by a resistance barrier or low conductance gap-junctions between atrial and nodal cells (13).

Despite these functional data indicating the existence of a step-delay in the AVN, the input to AVN is rather broadly distributed through transitional cells around the AVN from the fast pathway to INE and histological studies failed to find thin layers of connective tissue surrounding AVN as in the Purkinje fibers and the exact mechanisms of step-delay remains unknown. Importantly, the earliest sites of the AVN upstrokes dynamically change during Wenckebach, occasionally from the lower nodal location after a very long delay from the atrial upstroke (Figure 3C beat 4), raising questions whether this AVN upstroke is a result of conduction or automaticity. Although we cannot rule out potential concealed pathways in 3D that were missed with the current optical mapping system, this result may imply important roles of pacemaker activity of AVN cells in AVN conduction and delay. Similar shifts in leading AP upstroke have been observed in SA node (35–37), that may occur with a hierarchy of loosely coupled oscillators (38). Indeed, multiple studies of drug-induced or inherited AV block indicate the importance of pacemaker potential in regulating AVN conduction. Ivabradine, I_f_ blocker, has a high risk of creating an AV block in patients (39) and HCN4 channel knockout in mice caused AV block as well as severe bradycardia (40). Mutations in SCN5A associated with fast inactivation and lack of late Na^+^ can result in AV block (41). Cardiac ryanodine receptor (RyR2) knockout mice also exhibited bradycardia and secondary AV block, supporting the importance of AV nodal pacemaker activity in AV nodal conduction (42). Pacemaker activity may have an impact on regulating resting membrane potential of AV nodal cells that can inactivate or accelerate recovery of depolarizing ion channels such as I_Na_ or I_Ca_ for conduction. Alternatively, input from atrial AP may cause phase resetting of AVN cells to initiate AVN APs. The AV nodal AP can exhibit an overshooting hyperpolarization during the AP downstroke, a characteristic repolarization behavior of pacemaker cells. The delay in conduction can be considered as delays in entrainment of pacemaker cells. The Wenckebach phenomena are common in phase resetting and entrainment of pacemaker cells including the SA node. Further studies are warranted to understand the exact mechanisms of AV nodal delay and its pacemaker activity.

### Delay in PVJs

Downstream from the His-bundle, the Purkinje network branches into right and left bundles and are distributed in the subendocardium of the ventricles. The Purkinje cells are typically larger than ventricular myocytes and arranged in a parallel pattern. These cells are covered by a thin layer of connective tissues, which provides electrical insulation as well as mechanical support. The electrical coupling between Purkinje and Ventricular myocytes occurs at specialized Junctions (PVJs) (2). The structure of Purkinje network and the location of Purkinje-Ventricular Junctions (PVJs) are key elements for the stability and resilience of cardiac conduction system and the synchronization of ventricular contractions.

Previous experimental and clinical studies have shown that Purkinje network plays significant roles in arrhythmogenesis (43–51). The Purkinje myocytes can generate automaticity and triggered activity including early and delayed afterdepolarizations to initiate ventricular arrhythmias. The Purkinje network can accommodate reentrant circuits that can be stopped by an ablation procedure (52, 53). In addition, Purkinje fibers have been implicated in the transition from VT to VF and maintenance of VF, and ablations targeting certain regions of Purkinje fibers results in effective reduction of VF burden, suggesting important roles of Purkinje fibers in cardiac arrhythmias (53–55). In both cases, the PVJ has been implicated as a source of automaticity, triggered activity, and unidirectional block to form reentry circuits. This is mainly due to the cell-cell coupling across heterogeneous cell types and source-sink mismatch due to the small number of Purkinje cells connected to a large number of ventricular myocytes.

Conduction delay across PVJs were first investigated with multiple surface and microelectrodes. Pioneering work by Joyner's group reported activation delay between Purkinje cells and ventricular muscle cells at a junctional site characterized by transitional cells that exhibit double potentials (4, 56, 57). Importantly, these junctional complex can be found at discrete locations; some of Purkinje cells may form PVJs while others appear to be substantially uncoupled from neighboring cells (57). Here, optical mapping at high spatiotemporal resolution allows us to visualize differences in PVJ delays and how the PF network is involved delineating the sequence of papillary muscle activation followed by impulse propagation in the ventricular myocardium. The higher resistance across the PVJs is thought to protect the rapid propagation velocity along Purkinje fibers while retaining a safe conduction to ventricular myocytes by overcoming source-sink mismatch. Conduction safety and source-sink mismatch across PJV were studied mainly using computer modeling where small linear Purkinje fibers are connected to large 3D ventricular myocardium (5, 58–60). Computer modeling studies predicted that for safe impulse propagation, it is essential to have sufficient resistance between Purkinje and ventricular cells, which ranges from 3 to 5 ms conduction delay across PVJ. In this way, the depolarizing current does not quickly dissipate to the neighboring ventricular myocytes while triggering APs in ventricular myocytes. Our data detect a step-delay of 3–5 ms between two distinct upstrokes (Figure 3D), in line with previous studies with microelectrodes as well as computer modeling studies. Anatomical and microelectrode experiments have identified “transitional” cells separating PF from myocytes at PVJs. Transitional cells were described as thin, capacitor-like structures that electrically isolate PFs from direct electrical coupling with ventricular myocytes and may account for delays at PVJ (61). However, due to redundancies in PVJs, it is possible that the real conduction delay across PVJ can be longer than that measured here because ventricular activation in one PVJ can initiate AP propagation in the ventricular myocardium, which may result in seemingly shorter conduction delays in other PVJs (62). To determine the functional role of free-running PF, these fibers were severed which resulted in altered ventricular activation patterns and in conduction delays (Figure 6). This suggests that PVJ redundancy can shorten conduction delay and provides resilience and increases in the safety of conduction. Interestingly, our data also show longer step-delays ranging 5–15 ms. Typically, free-running PFs (false tendon) exhibited longer conduction delays but even in the trabecular muscle where PFs are embedded, the conduction delay is often longer than 5 ms. In this case, AP propagates rapidly across PFs without immediately triggering the neighboring ventricular myocytes (Figure 4A), suggesting that PFs may not form PVJs in this region. This design may allow rapid delivery of impulse to the remote region without slowing conduction through PVJs (4, 56, 58).

Heterogeneous refractoriness and conduction delay may underlie Purkinje-associated arrhythmogenesis. Different regions of PFs may have different APD and refractoriness can be heterogeneous. According to “gate theory” (53, 63, 64), heterogeneous refractoriness can cause conduction block in one PVJ but normal conduction across another PVJ (gate), causing unidirectional conduction to form reentry. We only investigated PV conduction on structurally normal hearts and PF-associated arrhythmias were not observed. Fast pacing at the right atrial appendage did not cause heterogeneous PF propagation. Due to redundancy in PVJ network, it is difficult to determine if conduction delays at certain location are true conduction delays from Purkinje-ventricular coupling. APD measurements from PFs were not possible with our current optical apparatus because the plateau and repolarization phase of PF action potentials are obscured by the long ventricular AP. Further studies with narrow depth-of-field or optogenetic probes to target Purkinje network may allow mapping of PF repolarization and refractoriness to highlight PF-related arrhythmias.

In conclusion, our data demonstrated that AP propagation through specialized cardiac conduction system can be mapped with high spatial and temporal optical mapping. Step-delays in conduction systems play significant roles in maintaining robust and safe impulse propagation, and their heterogeneity may underlie conduction diseases and arrhythmogenesis.

## Data Availability

The raw data supporting the conclusions of this article will be made available by the authors, without undue reservation.
